# Ribosomal binding site sequences and promoters for expressing glutamate decarboxylase and producing γ-aminobutyrate in *Corynebacterium glutamicum*

**DOI:** 10.1186/s13568-018-0595-2

**Published:** 2018-04-18

**Authors:** Feng Shi, Mingyue Luan, Yongfu Li

**Affiliations:** 1 State Key Laboratory of Food Science and Technology, Jiangnan University, 1800 Lihu Avenue, Wuxi, 214122 China; 2 Key Laboratory of Industrial Biotechnology, Ministry of Education, School of Biotechnology, Jiangnan University, Wuxi, 214122 China; 3 International Joint Laboratory on Food Safety, Jiangnan University, Wuxi, 214122 China; 4 National Engineering Laboratory for Cereal Fermentation Technology, Jiangnan University, Wuxi, 214122 China

**Keywords:** Ribosomal binding site (RBS), Promoter, Glutamate decarboxylase, γ-Aminobutyric acid, *Corynebacterium glutamicum*, *gadB2*

## Abstract

Glutamate decarboxylase (GAD) converts l-glutamate (Glu) into γ-aminobutyric acid (GABA). *Corynebacterium glutamicum* that expresses exogenous GAD gene, *gadB2* or *gadB1*, can synthesize GABA from its own produced Glu. To enhance GABA production in *C. glutamicum*, ribosomal binding site (RBS) sequence and promoter were searched and optimized for increasing the expression efficiency of *gadB2*. R4 exhibited the highest strength among RBS sequences tested, with 6 nt the optimal aligned spacing (AS) between RBS and start codon. This combination of RBS sequence and AS contributed to *gadB2* expression, increased GAD activity by 156% and GABA production by 82% compared to normal strong RBS and AS combination. Then, a series of native promoters were selected for transcribing *gadB2* under optimal RBS and AS combination. P_*dnaK*_, P_*dtsR*_, P_*odhI*_ and P_*clgR*_ expressed *gadB2* and produced GABA as effectively as widely applied P_*tuf*_ and P_*cspB*_ promoters and more effectively than P_*sod*_ promoter. However, each native promoter did not work as well as the synthetic strong promoter P_*tacM*_, which produced 20.2 ± 0.3 g/L GABA. Even with prolonged length and bicistronic architecture, the strength of P_*dnaK*_ did not enhance. Finally, *gadB2* and mutant *gadB1* were co-expressed under the optimal promoter and RBS combination, thus converted Glu into GABA completely and improved GABA production to more than 25 g/L. This study provides useful promoters and RBS sequences for gene expression in *C. glutamicum*.

## Introduction

γ-Aminobutyric acid (GABA) is a non-protein amino acid widely synthesized by diverse microorganisms, plants and animals (Li and Cao 2010; Shi et al. 2016). It functions as the major inhibitory neurotransmitter for the mammalian central nervous system and has several therapeutic abilities including hypotension, anti-anxiety, anti-depression, anti-schizophrenia, diuresis and anti-obesity (Wong et al. 2003; Mohler 2012; Shi et al. 2016). Therefore, GABA has been used as a bioactive component in functional foods and feeds. In addition, GABA is a crucial building block for the synthesis of bio-plastics, such as the biodegradable polyamide nylon 4 (Park et al. 2013).

GABA is mainly converted from l-glutamate (Glu) by lactic acid bacteria (LAB) and *Escherichia coli* strains with high Glu decarboxylase (GAD) activity (Li and Cao 2010; Shi et al. 2016). Although production of GABA by LAB, such as *Lactobacillus brevis* (Shi et al. 2017b; Wu et al. 2017), is recognized as safe, the cost of production is high; whereas production of GABA by *E. coli* is high yield (Huang et al. 2016; Zhao et al. 2016a) but is not acceptable for food usage. Furthermore, Glu must be added as the precursor during GABA production by LAB and *E. coli*, making such bioconversion not cost-effective. *Corynebacterium glutamicum* is generally regarded as safe and is widely used for the industrial production of Glu, l-lysine (Lys) and other amino acids (Leuchtenberger et al. 2005; Schneider et al. 2011). Recently, two metabolic routes for de novo biosynthesis of GABA from glucose have been employed in *C. glutamicum*, both using its own produced Glu as precursor. One route is fulfilled through multi-step reactions via putrescine (Jorge et al. 2016, 2017), the other only involves one-step reaction catalyzed by GAD (Okai et al. 2014; Choi et al. 2015; Shi et al. 2017a). Therefore, the expression level and activity of GAD is the main determinant for high-yield production of GABA through the second route.

The expression level of a protein is determined by transcription, translation and degradation rates, among them the beginning of transcription and translation are usually the rate-limiting steps. The initiation of transcription is fundamentally controlled by the promoter elements, while the initiation of translation is mainly affected by the strength of ribosomal binding site (RBS). Therefore, optimization of promoters and RBS sequences has been applied for protein expression in *C. glutamicum*. For example, *aph* promoter combined with different RBS sequences were used for expression of reporter proteins in *C. glutamicum* (Zhang et al. 2017b). Combinatorial assembly of *tac*, *cspB* and *sod* promoters and *lacZ*, *cspB* and *sod* RBS elements conferred differential expression of two reporters, eGFP and mCherry in pTGR platform of *C. glutamicum* (Ravasi et al. 2012). RBS sequence is also recorded as Shine–Dalgarno (SD) sequence in bacteria. The expression of GFP was enhanced in *C. glutamicum* using *tac* promoter and *tpi* SD sequence in the secretion vector (Teramoto et al. 2011).

Optimization of RBS sequence has also been applied for metabolic engineering of *C. glutamicum*. Through constructing RBS libraries and regulating the pathway genes *aroG*, *aroB*, *aroD* and *aroE* by RBS of different strengths, 9 genetic modules were built up and shikimic acid synthesis was improved in *C. glutamicum* (Zhang et al. 2015a). After replacing the compressed RBS of *vio* genes with complete strong *C. glutamicum* RBS and altering gene order to form a novel *vio* operon, hyper production of violacein was achieved in *C. glutamicum* (Sun et al. 2016).

Because promoter is essential for gene transcription, series of promoters for gene expression in *C. glutamicum* have been studied. The chimeric *tac* promoter and its modified *tacM* promoter are the most commonly used promoters in expression plasmids of *C. glutamicum*. In addition, several native promoters of *C. glutamicum*, such as the strong constitutive promoters of P_*sod*_, P_*tuf*_ and P_*cspB*_ (Ravasi et al. 2012; Lee 2014; Man et al. 2016) and the inducible promoter of P_*prpD2*_ (Pátek et al. 2013; Plassmeier et al. 2013), have been applied for modulating gene expression and for metabolic engineering of *C. glutamicum*. To obtain more promoters with different activities, synthetic promoters were recently isolated or constructed for gene expression in *C. glutamicum* (Yim et al. 2013; Rytter et al. 2014; Choi et al. 2015; Shen et al. 2017). Delightfully, the genetic elements including promoter regions and RBSs of nearly all the genes of *C. glutamicum* ATCC 13032 have been successfully identified based on an improved RNAseq technique (Pfeifer-Sancar et al. 2013), thus contribute to the analysis and usage of more and more native promoters.

It is reported that the initial translation efficiency and expression level of different proteins through the identical RBS sequence is dramatically different (Salis et al. 2009). To enhance the translation of GAD and production of GABA in recombinant *C. glutamicum*, a series of RBS sequence and aligned spacing (AS) were explored here for expressing a GAD gene, i.e. *gadB2* derived from *L. brevis* Lb85, by *tacM* promoter. Then, to improve the transcription of *gadB2* and yield of GABA, several types of native promoters and various architecture of the strong native promoter were explored. Considering the beneficial effects of *gadB1*–*gadB2* co-expression (Shi et al. 2013) as well as the mutant *gadB1*^T17I/D294G/E312S/Q346H^ expression (Shi et al. 2014) on GABA production, co-expression of *gadB1*^mut^ and *gadB2* was researched at last when the most effective RBS and promoter was verified.

## Materials and methods

### Strains, media, and growth conditions

Bacterial strains used in this study are listed in Table 1. *E. coli* JM 109 was employed as the host for constructing and propagating the plasmids. *E. coli* was grown in Luria–Bertani (LB) medium at 37 °C and 200 rpm. *C. glutamicum* SH, an l-glutamate-producing strain, was used for expressing *gadB2* and *gadB1* genes and producing GABA. SH was deposited in the China General Microbiological Culture Collection (CGMCC) center with accession number CGMCC 1.581. *C. glutamicum* was grown in LBG medium (LB supplemented with 5 g/L glucose) at 200 rpm and 30 °C. When necessary, 30 μg/mL kanamycin was added to the media.

**Table 1:** Strains use in this study

Strains	Characteristics	Source
*E. coli* JM 109	*E. coli* gene cloning strain	Novagen
*C. glutamicum* SH	Wild type *C. glutamicum*	CGMCC
R-B2	*C. glutamicum* SH harbouring pJYW-5-*gadB2*	This work
R1-B2	*C. glutamicum* SH harbouring pJYW-5-R1-*gadB2*	This work
R2-B2	*C. glutamicum* SH harbouring pJYW-5-R2-*gadB2*	This work
R3-B2	*C. glutamicum* SH harbouring pJYW-5-R3-*gadB2*	This work
R4-B2	*C. glutamicum* SH harbouring pJYW-5-R4-*gadB2*	This work
R5-B2	*C. glutamicum* SH harbouring pJYW-5-R5-*gadB2*	This work
R6-B2	*C. glutamicum* SH harbouring pJYW-5-R6-*gadB2*	This work
R7-B2	*C. glutamicum* SH harbouring pJYW-5-R7-*gadB2*	This work
R8-B2	*C. glutamicum* SH harbouring pJYW-5-R8-*gadB2*	This work
R9-B2	*C. glutamicum* SH harbouring pJYW-5-R9-*gadB2*	This work
R10-B2	*C. glutamicum* SH harbouring pJYW-5-R10-*gadB2*	This work
R11-B2	*C. glutamicum* SH harbouring pJYW-5-R11-*gadB2*	This work
R12-B2	*C. glutamicum* SH harbouring pJYW-5-R12-*gadB2*	This work
R4a-B2	*C. glutamicum* SH harbouring pJYW-5-R4a-*gadB2*	This work
R4b-B2	*C. glutamicum* SH harbouring pJYW-5-R4b-*gadB2*	This work
P*sod*-B2	*C. glutamicum* SH harbouring pJYW-5-P_*sod*_-R4a-*gadB2*	This work
P*uspA*-B2	*C. glutamicum* SH harbouring pJYW-5-P_*uspA*_-R4a-*gadB2*	This work
P*cspB*-B2	*C. glutamicum* SH harbouring pJYW-5-P_*cspB*_-R4a-*gadB2*	This work
P*tuf*-B2	*C. glutamicum* SH harbouring pJYW-5-P_*tuf*_-R4a-*gadB2*	This work
P*gdh*-B2	*C. glutamicum* SH harbouring pJYW-5-P_*gdh*_-R4a-*gadB2*	This work
P*dtsR*-B2	*C. glutamicum* SH harbouring pJYW-5-P_*dtsR*_-R4a-*gadB2*	This work
P*odhI*-B2	*C. glutamicum* SH harbouring pJYW-5-P_*odhI*_-R4a-*gadB2*	This work
P*sigB*-B2	*C. glutamicum* SH harbouring pJYW-5-P_*sigB*_-R4a-*gadB2*	This work
P*hmp*-B2	*C. glutamicum* SH harbouring pJYW-5-P_*hmp*_-R4a-*gadB2*	This work
P*pqo*-B2	*C. glutamicum* SH harbouring pJYW-5-P_*pqo*_-R4a-*gadB2*	This work
P*gapA*-B2	*C. glutamicum* SH harbouring pJYW-5-P_*gapA*_-R4a-*gadB2*	This work
P*ilvE*-B2	*C. glutamicum* SH harbouring pJYW-5-P_*ilvE*_-R4a-*gadB2*	This work
P*cg1417*-B2	*C. glutamicum* SH harbouring pJYW-5-P_*cg1417*_-R4a-*gadB2*	This work
P*dnaK*-B2	*C. glutamicum* SH harbouring pJYW-5-P_*dnaK*_-R4a-*gadB2*	This work
P*clgR*-B2	*C. glutamicum* SH harbouring pJYW-5-P_*clgR*_-R4a-*gadB2*	This work
P*clpB*-B2	*C. glutamicum* SH harbouring pJYW-5-P_*clpB*_-R4a-*gadB2*	This work
P*dnaJ*-B2	*C. glutamicum* SH harbouring pJYW-5-P_*dnaJ*_-R4a-*gadB2*	This work
P*sufR*-B2	*C. glutamicum* SH harbouring pJYW-5-P_*sufR*_-R4a-*gadB2*	This work
P*trxB1*-B2	*C. glutamicum* SH harbouring pJYW-5-P_*trxB1*_-R4a-*gadB2*	This work
P*dnaK*2SD-B2	*C. glutamicum* SH harbouring pJYW-5-P_*dnaK*_-2SD-*gadB2*	This work
P*dnaK*(+1)-B2	*C. glutamicum* SH harbouring pJYW-5-P_*dnaK*(+1)_-R4a-*gadB2*	This work
P*dnaK*(−1)-B2	*C. glutamicum* SH harbouring pJYW-5-P_*dnaK*(−1)_-R4a-*gadB2*	This work
P*dnaK*(−2)-B2	*C. glutamicum* SH harbouring pJYW-5-P_*dnaK*(−2)_-R4a-*gadB2*	This work
P*dnaK*(−3)-B2	*C. glutamicum* SH harbouring pJYW-5-P_*dnaK*(−3)_-R4a-*gadB2*	This work
R4a-B1^mut^	*C. glutamicum* SH harbouring pDXW-10-R4a-*gadB1*^mut^	This work
R4a-B2B1^mut^	*C. glutamicum* SH harbouring pJYW-5-R4a-*gadB2*-R4a-*gadB1*^mut^	This work

### Construction of *gadB2* expression strains under different RBS sequence and promoter

The nucleotide sequences of all primers are listed in Table 2. A series of RBS sequence derived from the consensus sequence AGGAG and an AS of 6–8 nt was designed and applied for expressing *gadB2*. For expressing with normal RBS sequence and AS of 7 nt, *gadB2* was amplified from the plasmid pJYW-4-*gadB1*–*gadB2* (Shi et al. 2017a) using the primer pair of R-B2-F and gadB2-R. The PCR product was digested with *Hpa*I and *Bam*HI, and ligated into pJYW-5, a *E. coli*–*C. glutamicum* shuttle expression vector carrying a *tacM* promoter, resulting in the plasmid pJYW-5-*gadB2.* For expressing with the 12 designed RBS sequence (R1–R12) and AS of 7 nt as well as with RBS of R4 and AS of 6 nt (R4a) or 8 nt (R4b), *gadB2* was amplified similarly using the 14 different forward primer Rn-B2-F and the same reverse primer gadB2-R, digested with *Afl*II and *Bam*HI, and ligated into pJYW-5 digested with the same enzymes, resulting in the 14 plasmids of pJYW-5-Rn-*gadB2*. Finally, these plasmids were transformed into *C. glutamicum* SH by the method described previously (Wang et al. 2015), generating 15 recombinant strains of R-B2, R1-B2 to R12-B2, R4a-B2 and R4b-B2.

**Table 2:** Primers used in this study

Primers	Sequences (5′–3′)	Restriction sites
R-B2-F	GGGGTTAAC*AGAAGGAG***GGATTGC**ATGAATAAAAACGATCAGGA	*Hpa*I
gadB2-R	CGGGATCCTTAACTTCGAACGGTGGTCT	*Bam*HI
R1-B2-F	CGGGGCTTAAG*AAAAGGAG***GGATTGC**ATGAATAAAAACGATCAGGA	*Afl*II
R2-B2-F	CGGGCTTAAG*GGAAGGAG***GGATTGC**ATGAATAAAAACGATCAGGA	*Afl*II
R3-B2-F	GCCCTTAAG*GAAAGGCGA***GGATTGC**ATGAATAAAAACGATCAGGA	*Afl*II
R4-B2-F	CGGGGCTTAAG*GAAAGGAGA***GGATTGC**ATGAATAAAAACGATCAGGA	*Afl*II
R5-B2-F	CGGGCTTAAG*GAAAGGAG***GGATTGC**ATGAATAAAAACGATCAGGA	*Afl*II
R6-B2-F	CGGGCTTAAG*AGGAGGAG***GGATTGC**ATGAATAAAAACGATCAGGA	*Afl*II
R7-B2-F	CGGGGCTTAAG*GAAAGGA***GGATTGC**ATGAATAAAAACGATCAGGA	*Afl*II
R8-B2-F	CGGGGCTTAAG*AGAAAGGAGG***GGATTGC**ATGAATAAAAACGATCAGGA	*Afl*II
R9-B2-F	CGGGGCTTAAG*GAAAGGAGG***GGATTGC**ATGAATAAAAACGATCAGGA	*Afl*II
R10-B2-F	GGCGCTTAAG*GAGAGGAG***GGATTGC**ATGAATAAAAACGATCAGGA	*Afl*II
R11-B2-F	CGGGGCTTAAG*AAGAGGAG***GGATTGC**ATGAATAAAAACGATCAGGA	*Afl*II
R12-B2-F	CGGGGCTTAAG*GGGAGGAG***GGATTGC**ATGAATAAAAACGATCAGGA	*Afl*II
R4a-B2-F	GCCGGCTTAAG*GAAAGGAGA***GGATTG**ATGAATAAAAACGATCAGGA	*Afl*II
R4b-B2-F	GCCGGCTTAAG*GAAAGGAGA***GGATTGCA**ATGAATAAAAACGATCAGGA	*Afl*II
R4a-B1^mut^-F	GGAAGATCT*GAAAGGAGA***GGATTG**ATGGCTATGTTGTATGGAA	*Bgl*II
gadB1^mut^-R	AACTGCAGTTAGTGCGTGAACCCGTATTTTTT	*Pst*I
M-R4a-B1^mut^-F	ACGCGTCGACGGTTCTGGCAAATATTCTGAAA	*Sal*I
P_*sod*_-F	CTAGTCTAGACTTATGCCCTTCAACCCTACTTA	*Xba*I
P_*sod*_-R	GCAAGATCTAGATTCGTAGGTTTCCGCACCGAGCATA	*Xba*I
P_*uspA*_-F	CTAGTCTAGACGAGATGAGATCTTCGAGCT	*Xba*I
P_*uspA*_-R	GCGGACTCTAGAAAACTTATGACAGGGGTTAAAA	*Xba*I
P_*cspB*_-F	CTAGTCTAGATAGTCAAGAATTTACCCCCT	*Xba*I
P_*cspB*_-R	CTAGTCTAGATTGAACACTGCGCACTGAAA	*Xba*I
P_*tuf*_-F	CATTTCTAGATAAGTGGGGTAGCGGCTTGT	*Xba*I
P_*tuf*_-R	CCGCTATCTAGAAATTGGTTTTGCTTTCACTG	*Xba*I
P_*gdh*_-F	CCTAGTCTAGAGAAGAGACTTCATGCAGTTACC	*Xba*I
P_*gdh*_-R	CCGGTCTAGAACGATTTTAAAGTGTGTATCTG	*Xba*I
P_*dtsR*_-F	CTAGTCTAGACACGCCCAAAAAGTTTTACC	*Xba*I
P_*dtsR*_-R	CCTAGTCTAGATGTTTTGAAATCGTAGCGGTA	*Xba*I
P_*odhI*_-F	CATTTCTAGACGATCACGAGGGGGCACATT	*Xba*I
P_*odhI*_-R	CCCGGCTCTAGATCTTAACGATTTTCATCATA	*Xba*I
P_*sigB*_-F	ATATTCTAGATTAACGAAGGCCCCTTCACC	*Xba*I
P_*sigB*_-R	GTATTCTAGAGGTTCAAGAGGTTCAACGGA	*Xba*I
P_*hmp*_-F	CTCCTCTAGACGATAAAACGGCATTAAACC	*Xba*I
P_*hmp*_-R	CGGGTCTAGAGCTAATTTCTACGGATTTAATC	*Xba*I
P_*pqo*_-F	GTCCTCTAGAAGCAACGACGGAAATCCCAAAA	*Xba*I
P_*pqo*_-R	GTTCTCTAGATGCCTAACTTGGTGCGACTT	*Xba*I
P_*gapA*_-F	GCCATCTAGAAAACTATTTAGCGCAAGTGT	*Xba*I
P_*gapA*_-R	GCCATCTAGATGTAGGAAATGCAATGTGTC	*Xba*I
P_*ilvE*_-F	ATACTCTAGAGGGGGAGGCATCAAATTGA	*Xba*I
P_*ilvE*_-R	GCGCTCTAGATCATGGATTTTAAGGTACAC	*Xba*I
P_*cg1417*_-F	GTCCTCTAGAGTGTCGTTTTTTGTGCACCTGTTTTG	*Xba*I
P_*cg1417*_-R	GGGGTCTAGAGAAAATCCAGGTTAAAAGC	*Xba*I
P_*dnaK*_-F	CGCGTCTAGAATTGGGTGGTTGAAAATTAG	*Xba*I
P_*dnaK*_-R	CGCGTCTAGAGTGATTTTAGTACTGTCCA	*Xba*I
P_*clgR*_-F	ATATTCTAGAAACAAGATGGTCATCCGGTG	*Xba*I
P_*clgR*_-R	GCTTTCTAGACGTTAGGTTCAACTCCCTTT	*Xba*I
P_*clpB*_-F	ATATTCTAGATGATTTTTGGCCTCGCGTGG	*Xba*I
P_*clpB*_-R	CGATTCTAGAGCCAACCTACACAATCAGT	*Xba*I
P_*dnaJ*_-F	CATTTCTAGATTAGTGGTTTCCGCCGTTGT	*Xba*I
P_*dnaJ*_-R	CAGGTCTAGAGAGTCTTATATGCGGTGAAT	*Xba*I
P_*sufR*_-F	CTAGTCTAGATACCTTTGGTTGGCTTAGGG	*Xba*I
P_*sufR*_-R	CTAGTCTAGATGGTGTCACCTCCTGCTTGA	*Xba*I
P_*trxB1*_-F	CTAGTCTAGACCGCAACAATGCCGATTTCA	*Xba*I
P_*trxB1*_-R	CGCGTCTAGATTCAAGATTTGTAAGGTCTA	*Xba*I
P_*dnaK*_-2SD-R	CAT**TAATCC***TCTCCTTTC*GAGTTGGTGGTTCCAAGGTCA	
P_*dnaK*_-2SDB2-F	CCACCAACTC*GAAAGGAGA***GGATTA**ATGAATAAAAACGATCAGGAAACACAGC	
P_*dnak*(+1)_-R	CAT**CAATCC***TCTCCTTTC*AGTTGGTGGTTCCAAGGTCA	
P_*dnaK*(−1)_-F	ATTACTCTAGAGCGTGAGACTTGGTGTCAAA	*Xba*I
P_*dnaK*(−2)_-F	ATTACTCTAGAGGATGGTGCTTTTTGCATC	*Xba*I
P_*dnaK*(−3)_-F	ATATCTCTAGAGCGGTGGCTCAAAATTGCCTTCA	*Xba*I
P_*dnaK*(−)_-R	CAT**CAATCC***TCTCCTTTC*GTGATTTTAGTACTGTCCAC	
P_*dnaK*()_B2-F	*GAAAGGAGA***GGATTG**ATGAA	
RT-*B2*-F	ACACAGGCTCCGTTGATGAT	
RT-*B2*-R	TTATGGCCCGAAACGTTAAT	
RT-16S-F	ACCTGGAGAAGAAGCACCG	
RT-16S-R	TCAAGTTATGCCCGTATCG	

The restriction sites are underlined. The RBSs are italicized. The ASs are in boldface

Each promoter sequence was designed according to the predicted promoter (Pfeifer-Sancar et al. 2013) with its upstream 60 bp. For obtaining different native promoters, P_*sod*_ and P_*tuf*_ were amplified from the genomic DNA of *C. glutamicum* ATCC 13032, while others were amplified from the genomic DNA of *C. glutamicum* SH using the corresponding primer pairs. To express *gadB2* with these promoters, the P_*tacM*_ was deleted from pJYW-5-R4a-*gadB2* at first; then, the PCR products of P_*sod*_, P_*uspA*_, P_*cspB*_, P_*tuf*_, P_*gdh*_, P_*dtsR*_, P_*odhI*_, P_*sigB*_, P_*hmp*_, P_*pqo*_, P_*gapA*_, P_*ilvE*_, P_*cg1417*_, P_*dnaK*_, P_*clgR*_, P_*clpB*_, P_*dnaJ*_, P_*sufR*_ and P_*trxB1*_ were digested with *Xba*I and ligated into the P_*tacM*_-deleted pJYW-R4a-*gadB2*, resulting in 19 plasmids of pJYW-5-P_*X*_-R4a-*gadB2*. Finally, these plasmids were transformed into *C. glutamicum* SH, generating 19 recombinant strains (from P*sod*-B2 to P*trxB1*–B2 in Table 1).

For expressing *gadB2* with bicistronic expression cassette (Zhao et al. 2016b), P_*dnaK*_-2SD-*gadB2* containing the first 41 bp of *dnaK* gene and the strong RBS (R4a) was amplified via overlap PCR using primers P_*dnaK*_-F, P_*dnaK*_-2SD-R, P_*dnaK*_-2SDB2-F and gadB2-R, and then ligated into pJYW-5, resulting in the plasmid pJYW-5-P_*dnaK*_-2SD-*gadB2*. To express *gadB2* with prolonged P_*dnaK*_, P_*dnaK*_ with the downstream 60 bp [P_*dnaK*(+1)_], with the upstream 60 bp [P_*dnaK*(−1)_], upstream 180 bp [P_*dnaK*(−2)_] and upstream 240 bp [P_*dnaK*(−3)_] were amplified with corresponding primers and then overlapped with R4a-*gadB2* fragment that was amplified with primers P_*dnaK*()_B2-F and gadB2-R, generating plasmids pJYW-5-P_*dnaK*(+1)_-R4a-*gadB2*, pJYW-5-P_*dnaK*(−1)_-R4a-*gadB2*, pJYW-5-P_*dnaK*(−2)_-R4a-*gadB2* and pJYW-5-P_*dnaK*(−3)_-R4a-*gadB2*. Finally, the 5 plasmids were transformed into *C. glutamicum* SH, yielding 5 recombinant strains, P*dnaK*2SD-B2, P*dnaK*(+1)-B2, P*dnaK*(−1)-B2, P*dnaK*(−2)-B2 and P*dnaK*(−3)-B2.

### Construction of *gadB1*^mut^ expression strain and *gadB2*–*gadB1*^mut^ co-expression strain

*gadB1*^mut^ gene with the strong RBS (R4a) was amplified from the plasmid pEC-*gadB1*^T17I/D294G/E312S/Q346H^ (Shi et al. 2014) using the primer pair of R4a-B1^mut^-F and gadB1^mut^-R. The PCR product was digested with *Bgl*II and *Pst*I, and ligated into pDXW-10, a shuttle expression vector between *E. coli* and *Corynebacteria* which carries a *tacM* promoter (Xu et al. 2010), resulting in the plasmid pDXW-10-R4a-*gadB1*^mut^. Then the DNA fragment containing P_*tac*M_ and R4a-*gadB1*^mut^ was amplified from pDXW-10-R4a-*gadB1*^mut^ using primer pair of M-R4a-B1^mut^-F and gadB1^mut^-R, digested with *Sal*I and *Pst*I, and ligated into pJYW-5-R4a-*gadB2* that was similarly digested, resulting in the plasmid pJYW-5-R4a-*gadB2*-R4a-*gadB1*^mut^. The two plasmids were finally transformed into *C. glutamicum* SH, generating recombinant strains R4a-B1^mut^ and R4a-B2B1^mut^.

### GABA fermentation of recombinant *C. glutamicum* strains in shake flask

For GABA production in a shake flask, recombinant *C. glutamicum* cells were pre-cultured in seed medium at 30 °C and 200 rpm for 9 h, inoculated into a 500-mL baffled flask containing 25 mL of fermentation medium to a final optical density (OD_562_) of 1.9 and cultured at 30 °C and 200 rpm for 72 h as previously described (Shi et al. 2013) by a cyclotron shaker. At 10, 11.5, 13, 14.5, 18 and 21.5 h of fermentation, 2 g/L urea was added to the culture to maintain the neutral conditions. After 24 h of fermentation, an appropriate volume of culture broth was harvested every 12 h, and the cell concentration, pH, residual glucose concentration and the Glu and GABA concentrations in the fermentation broth were measured by the method described previously (Shi et al. 2013).

### Assay of GAD activity

The cells in the fermentation broth were harvested and washed twice with chilled phosphate buffer saline. However, no GAD activity could be detected after the crude enzyme was extracted from washed cells by method described previously (Shi et al. 2013). Therefore, the washed cells were re-suspended in equal volume of 0.02 M Na_2_HPO_4_–citric acid buffer (pH 4.8) containing 10% glycerol and 0.1% Triton, and applied directly as the crude enzyme of whole cell suspension. The GAD reaction was then performed at 37 °C for 1 h in a reaction mixture (1 mL) consisting of 0.4 M Na_2_HPO_4_–citric acid buffer (pH 5.0), 60 mM monosodium glutamate, 0.03 mM pyridoxal 5′-phosphate and appropriate volume of whole cell suspension. GAD activity was determined according to the formation of GABA in this reaction. One unit (U) of GAD activity is defined as 1.0 µmol GABA produced in 1 min in the initial reaction mixture. The specific activity is expressed as U/g of dry cell weight (DCW). The DCW per liter (g/L) was calculated according to an experimentally determined formula: DCW = 0.6495 × OD_562_ − 2.7925.

### Real-time PCR analysis of *gadB2* transcription

The mRNA transcription levels of *gadB2* gene in recombinant *C. glutamicum* during fermentation were determined by real-time PCR (RT-PCR) combined with reverse transcription as described previously (Wang et al. 2015). Total RNA was extracted from cells that were harvested at 20 and 40 h. After disposed DNA with DNase I, the quality and amount of RNA were analyzed and quantified by electrophoresis. Then the mRNAs were reverse transcribed into cDNAs and the cDNAs were used for RT-PCR analysis. Primers for RT-PCR are listed in Table 2. The relative abundance of *gadB2* mRNAs was quantified based on the cycle threshold (C_t_) value and was calculated by the $2^{{ - \Delta \Delta C_{t} }}$ method (Livak and Schmittgen 2001). To standardize the transcription levels, the relative abundance of 16S rRNA was used as the internal standard.

## Results

To improve GAD expression and GABA production in recombinant *C. glutamicum* SH, the RBS sequences and promoters for expressing GAD gene were explored here. As expression of *gadB2* produced more GABA in *C. glutamicum* than expression of *gadB1* (Shi and Li 2011), *gadB2* was selected for engineering of RBS sequence and promoter.

### Optimization of RBS sequence for *gadB2* expression and GABA production

RBS is a pivotal region for controlling translation initiation and protein expression. For expressing target protein(s) in *C. glutamicum*, AGAAGGAG was used as normal RBS sequence in our previous studies. However, some conserved RBS sequences, such as AGAAAGGAGG (Amador et al. 1999) and GAAAGGAGG (Martín et al. 2003), have been reported in *C. glutamicum*. In addition, several sequences such as GAAAGGAGA, GAAAGGCGA and GAAAGGA were used as strong RBS for expressing target genes in *C. glutamicum* (Kang et al. 2014; Zhang et al. 2015b). Thus these 5 RBS sequences were analyzed here for *gadB2* expression. Recently, AGGag was detected as a conserved motif in about 92% of 5’-UTR sequences of the entire protein-coding genes in *C. glutamicum* (Pfeifer-Sancar et al. 2013). Therefore, a seeding sequence of (A/G)_3_AGGAG was also synthesized here for expressing *gadB2*. The GAD activity and GABA production under these 12 new RBS sequences (R1–R12) were researched.

**Fig. 1:**
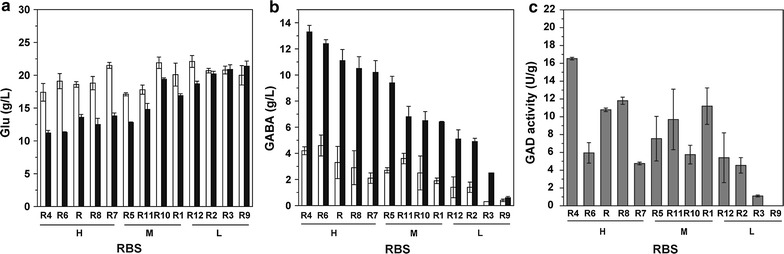
GABA production and GAD activity of *gadB2*-expressing *C. glutamicum* strains under different RBS sequences. **a** Glu concentration, **b** GABA concentration, **c** GAD activity. White bars at 36 h, black bars at 72 h, grey bars at 40 h. *H* high level of GABA, *M* medium level of GABA, *L* low level of GABA. Each point represents the average of three independent experiments

In recombinant *C. glutamicum*, the optimal pH for cell growth and Glu biosynthesis is about 7.0, whereas that for GAD activity and conversion of Glu to GABA is 5.0–6.0. For effective production of GABA, the cultivated medium was initially maintained at neutral pH by adding urea during 10–21.5 h of fermentation, and then the pH was let to decline and change spontaneously thereafter. During the fermentation, all the strains that harbor different RBS sequences grew similarly and exhibited similar pH variation. The cell density increased fast before 24 h and nearly maintained thereafter at OD_562_ of approximately 45–50. Glucose was consumed rapidly before 24 h, slowly thereafter and nearly exhausted at the end of fermentation. The pH value decreased to the lowest level of about 4.9–5.6 at 36 h, partially due to the exhaust of urea and accumulation of acidic Glu (Fig. 1a), and rose gradually to about 5.2–6.6 thereafter, partially due to the conversion of acidic Glu to neutral GABA (Fig. 1b). As pH decreased to the lowest level at 36 h, GAD became active and GABA began to synthesize quickly. Therefore, before 36 h was regarded as Glu fermentation stage and after 36 h was regarded as GABA conversion stage. It is worth mentioning that the yield of GABA was obviously different in *C. glutamicum* strains harboring different RBS sequences (Fig. 1b). Thus these recombinant strains were classified into three levels, high (H), medium (M) and low (L), according to their production capacity of GABA. In the H level, GABA production was higher than 10 g/L, with R4-B2 strain the highest (13.3 ± 0.5 g/L). The GABA production of R4-B2 was even higher than that of R-B2 under the normal RBS sequence. Meanwhile, less Glu was remained in these strains (Fig. 1a). However, GABA production decreased to about 6–10 g/L in the M level strains and even lower than 6 g/L in the L level strains. Furthermore, the GAD activity of H level strains was slightly higher than M level strains and obviously higher than L level strains, also with R4-B2 the highest (16.5 ± 0.2 U/g DCW) (Fig. 1c). Therefore, the translation of GadB2 was most efficient under the RBS sequence of R4.

### Optimization of aligned spacing for *gadB2* expression and GABA production

Even under the RBS sequence of R4, approximately 11 g/L of Glu was remained and not converted to GABA. Besides RBS sequence, aligned spacing between RBS and translational start codon is also important for translation efficiency. AS has been revealed to be 4–12 nt in *C. glutamicum*, with 6–8 nt as the most common (Pfeifer-Sancar et al. 2013). Therefore, the AS of 6–8 nt for RBS sequence of R4 was then analyzed for expressing *gadB2* and producing GABA.

**Fig. 2:**
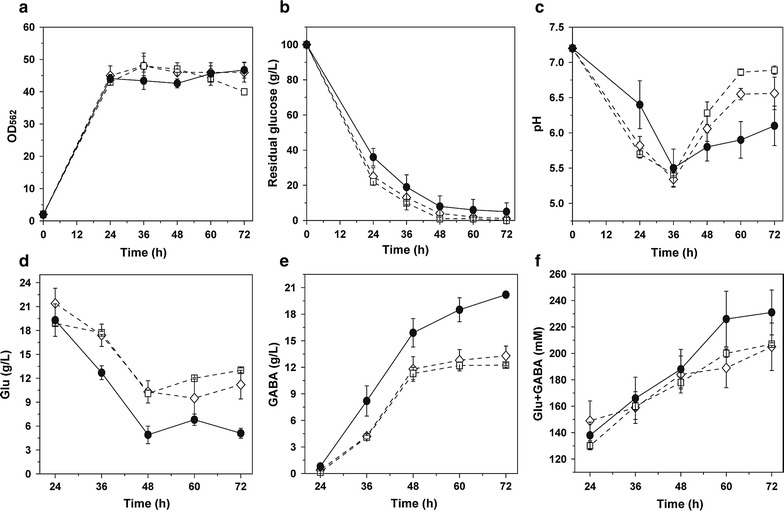
GABA fermentation by *gadB2*-expressing *C. glutamicum* strains under R4 RBS sequence with AS of 6 nt (R4a), 7 nt (R4) and 8 nt (R4b). **a** Cell growth, **b** glucose consumption, **c** pH variation, **d** Glu production, **e** GABA production, **f** total amount of Glu and GABA. Circles R4a-B2, empty diamonds in dotted line R4-B2, empty squares in dotted line R4b-B2. Averages of three independent experiments are provided

The three *gadB2*-expressing strains with different AS grew and consumed glucose similarly (Fig. 2a, b), but their pH value varied differently (Fig. 2c). The variation range of the pH of R4a-B2 with AS of 6 nt was less than that of R4-B2 with AS of 7 nt, whereas the pH range of R4b-B2 with AS of 8 nt was more than that of R4-B2. Most importantly, compared with R4-B2, significant more GABA (20.2 ± 0.3 g/L) was produced and less Glu (5.1 ± 0.6 g/L) was remained in R4a-B2, whereas in R4b-B2, same amount of GABA and Glu were produced (Fig. 2d, e). Meanwhile, the total amount of Glu and GABA was obviously high in R4a-B2 (Fig. 2f). In addition, the GAD activity of R4a-B2 (32.0 ± 2.5 U/g DCW) was significantly higher than R4-B2 (16.5 ± 0.2 U/g DCW) and R4b-B2 (21.4 ± 1.2 U/g DCW). Therefore, the AS of 6 nt with RBS of R4 (R4a) was more preferable than the AS of 7 nt and 8 nt for translation of GadB2 and production of GABA in *C. glutamicum*. Then R4a was selected as the most prominent combination of RBS sequence and AS thereafter.

### Different promoters for *gadB2* expression and GABA production

The translation efficiency of GadB2 was improved in R4a-B2, where *gadB2* was expressed by P_*tacM*_ promoter. P_*tacM*_ was derived from P_*tac*_ (Xu et al. 2010). P_*tac*_ was confirmed to be a strong promoter for expressing *lysE* and improving l-ornithine production (Rytter et al. 2014; Zhang et al. 2017a), and P_*tacM*_ was proven to be a stronger promoter than P_*tac*_ (Xu et al. 2010). Then the transcription of *gadB2* under the RBS and AS of R4a was researched and three kinds of native promoters for the transcription of *gadB2* were verified.

The first kind was widely used constitutive promoters (P_*sod*_, P_*tuf*_, P_*cspB*_ and P_*uspA*_) and several native promoters of genes involved in carbon metabolism (*dtsR*) and Glu biosynthesis (*gdh* and *odhI*). P_*sod*_ and P_*tuf*_ have been widely applied to enhance gene expression and optimize metabolic pathways for production of amino acids, such as l-ornithine (Kim et al. 2015), Lys (Becker et al. 2011; Shang et al. 2018) and l-araginine (Man et al. 2016) in *C. glutamicum*. P_*cspB*_ and P_*uspA*_ were used as strong promoters for expressing reporters in *C. glutamicum* (Ravasi et al. 2012; Zhao et al. 2016b). So these promoters were analyzed here for expressing *gadB2*.

**Fig. 3:**
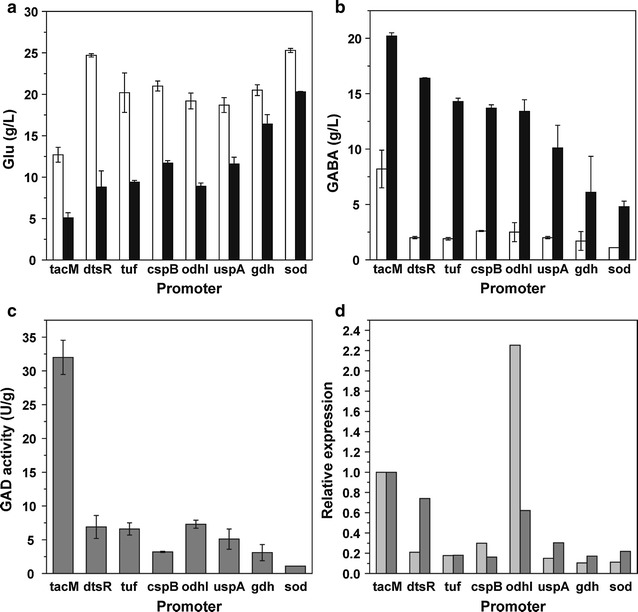
GABA production and expression level of *gadB2*-expressing *C. glutamicum* strains controlled by some constitutive promoters. **a** Glu concentration, **b** GABA concentration, **c** GAD activity, **d** *gadB2* transcription level. White bars at 36 h, black bars at 72 h, grey bars at 20 h, dark grey bars at 40 h. Each point represents the average of three independent experiments

The growth, glucose consumption and pH variation of recombinant *C. glutamicum* strains were not affected by the replacement of these 8 promoters, but the GABA production was quite different (Fig. 3b). R4a-B2, P*dtsR*-B2, P*tuf*-B2, P*cspB*-B2, P*odhI*-B2 and P*uspA*-B2 with P_*tacM*_, P_*dtsR*_, P_*tuf*_, P_*cspB*_, P_*odhI*_ and P_*uspA*_ promoter, respectively produced GABA more than 10 g/L, whereas P*gdh*-B2 with P_*gdh*_ promoter and P*sod*-B2 with P_*sod*_ promoter only produced about 5 g/L GABA. Unexpectedly, although the GABA production of P*dtsR*-B2 (16.4 ± 0.1 g/L) was significantly higher than that of strains under other native promoters, it was obviously lower than that of R4a-B2 under P_*tacM*_ promoter (20.2 ± 0.3 g/L) and more Glu was remained (Fig. 3a). Meanwhile, the GAD activity of P*gdh*-B2 and P*sod*-B2 (1.1–3.1 U/g DCW) was significantly lower than that of other strains (3.2–32.0 U/g DCW), especially R4a-B2 under P_*tacM*_ promoter (Fig. 3c), basically consistent with their GABA production. In addition, at Glu fermentation stage (20 h), only P*odhI*-B2 showed higher transcription level of *gadB2* than R4a-B2, whereas other strains showed significantly lower level than R4a-B2 (Fig. 3d). While at GABA conversion stage (40 h), P*dtsR*-B2 and P*odhI*-B2 showed slightly lower and other strains showed significantly lower transcription level of *gadB2* than R4a-B2. Therefore, these constitutive promoters and Glu synthesis-related promoters were not as effective as P_*tacM*_ for transcription of *gadB2*.

**Fig. 4:**
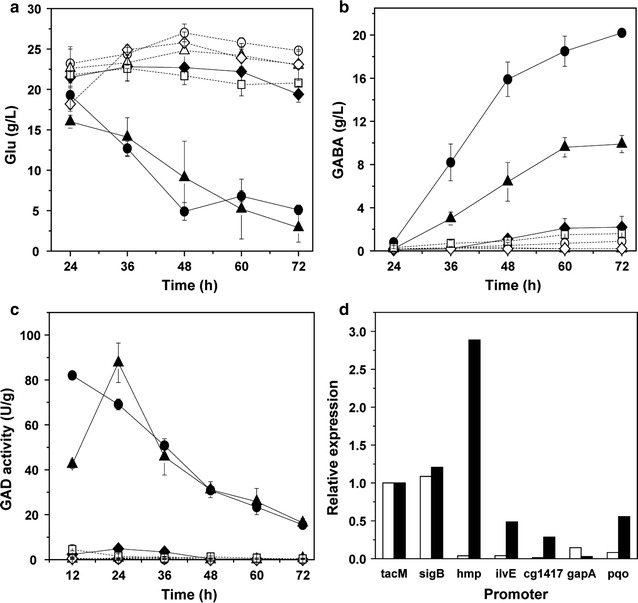
GABA fermentation of *gadB2*-expressing *C. glutamicum* strains controlled by *sigB* promoter and some σ^B^-recognized promoters. **a** Glu concentration, **b** GABA concentration, **c** GAD activity, **d** *gadB2* transcription level. Circles R4a-B2, triangles P*sigB*-B2, diamonds P*hmp*-B2, empty squares in dotted line P*pqo*-B2, empty circles in dotted line P*ilvE*-B2, empty diamonds in dotted line P*gapA*-B2, empty triangles in dotted line P*cg1417*-B2. White bars at 20 h, black bars at 40 h. Each point represents the average of three independent experiments

The second kind was *sigB* promoter and several σ^B^-recognized promoters, considering that GAD mainly acts at stationary phase when pH is below 6.0. Some promoters, such as P_*hmp*_, P_*pqo*_, P_*gapA*_, P_*ilvE*_ and P_*cg1417*_, had been proven to be σ^B^-dependent (Larisch et al. 2007; Pátek and Nešvera 2011). Then these promoters were analyzed here for expressing *gadB2*. During fermentation, most *gadB2*-expressing strains under σ^B^-related promoters grew and consumed glucose similarly with R4a-B2 under P_*tacM*_ promoter, except P*gapA*-B2 which grew and consumed glucose much slowly. Unfortunately, all the 5 σ^B^-recognized promoters tested here were far less robust for producing GABA (less than 3 g/L) and expressing GAD activity (less than 5 U/g DCW), although most of them except P_*gapA*_ were able to enhance the *gadB2* transcription level at GABA conversion stage (Fig. 4). P*sigB*-B2 showed comparable *gadB2* transcription level and GAD activity with R4a-B2; meanwhile, its GAD activity increased significantly during 12–24 h, whereas that of R4a-B2 decreased continuously during the whole fermentation. However, although the GABA production of P*sigB*-B2 was significantly higher than that of other 5 σ^B^-controlled strains, it was only half of that R4a-B2. Therefore, these σ^B^-related promoters were not effective for producing GABA.

**Fig. 5:**
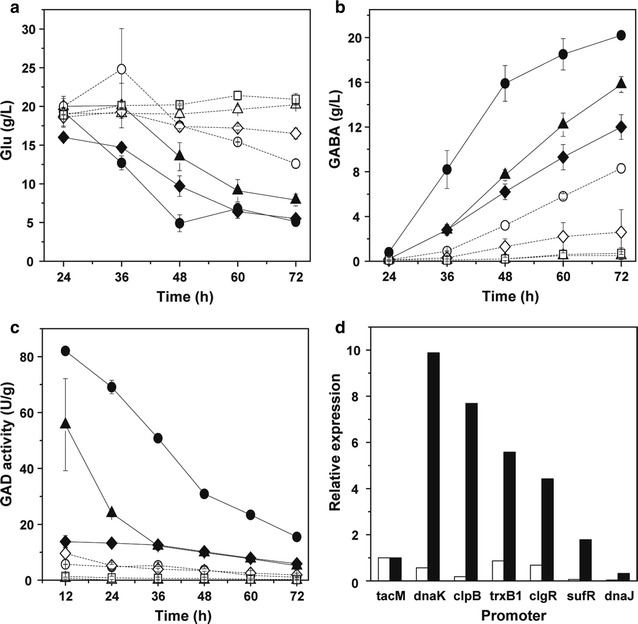
GABA fermentation of *gadB2*-expressing *C. glutamicum* strains controlled by some stress-response promoters. **a** Glu concentration, **b** GABA concentration, **c** GAD activity, **d** *gadB2* transcription level. Circles R4a-B2, triangles P*dnaK*-B2, diamonds P*clgR*-B2, empty circles in dotted line P*clpB*-B2, empty diamonds in dotted line P*trxB1*-B2, empty squares in dotted line P*dnaJ*-B2, empty triangles in dotted line P*sufR*-B2. White bars at 20 h, black bars at 40 h. Each point represents the average of three independent experiments

The third kind was several promoters of genes involved in stress response, because on account of transcriptome analysis, the expression of several stress response genes, i.e. *dnaK*, *clgR*, *clpB*, *trxB1*, *dnaJ* and *sufR*, up-regulated significantly during GABA conversion stage. Then, P_*dnaK*_, P_*clgR*_, P_*clpB*_, P_*trxB1*_, P_*sufR*_ and P_*dnaJ*_ were selected for expressing *gadB2*. The growth and glucose consumption of *gadB2*-expressing strains was not affected by the replacement of these stress response promoters, but the GABA production was quite different (Fig. 5b). P*dnaK*-B2 and P*clgR*-B2 accumulated GABA to more than 10 g/L, P*clpB*-B2 accumulated to approximately 8.0 g/L, whereas P*trxB1*-B2, P*dnaJ*-B2 and P*sufR*-B2 accumulated only less than 3 g/L. It was regrettable that although the GABA production of P*dnaK*-B2 (15.8 ± 0.7 g/L) was significantly higher than that of strains under other stress response promoters, it was obviously lower than that of R4a-B2 and more Glu was remained (Fig. 5a). In addition, the GAD activity of all strains decreased continuously during the whole fermentation, with R4a-B2 always exhibiting the highest activity, followed by P*dnaK*-B2 and P*clgR*-B2, whereas P*dnaJ*-B2 and P*sufR*-B2 nearly no activity (Fig. 5c). The GAD activity of these strains was basically consistent with their GABA production. Furthermore, at 20 h, P*trxB1*-B2, P*clgR*-B2 and P*dnaK*-B2 showed similar transcription level of *gadB2* to R4a-B2 and other 3 strains showed significantly lower level than R4a-B2 (Fig. 5d). While at 40 h, the *gadB2* transcription level of P*dnaK*-B2, P*clpB*-B2, P*trxB1*-B2, P*clgR*-B2 and P*sufR*-B2 was significantly higher than that of R4a-B2. Thus the transcription of *gadB2* was actually enhanced by these stress responsive promoters during GABA conversion stage. However, the GAD activity and GABA production did not improve accordingly, perhaps due to the translation and stability of GadB2 in these strains. Among all the three kinds of native promoters, P_*dnaK*_ seems to be most effective for *gadB2* expression and GABA production.

### Effect of length and architecture of P_*dnaK*_ on *gadB2* expression and GABA production

Although P_*dnaK*_ was the most effective native promoter for expressing *gadB2* and producing GABA, it was not as effective as P_*tacM*_. There are two transcription initiation sites in P_*dnaK*_, P1_*dnaK*_ promoter is recognized by σ^A^, P2_*dnaK*_ promoter is recognized by σ^E^ and σ^H^; meanwhile, P_*dnaK*_ is directly repressed by HspR (Ehira et al. 2009; Šilar et al. 2016). Therefore, cis-regulatory elements may be present in the flanking region of P_*dnaK*_. Then various length of P_*dnaK*_ with extended flanking sequence was tested for its strength. In addition, the coden sequence downstream of initiation coden, especially the following two codens was shown to be crucial for translational efficiency (Stenstrom et al. 2001). Based on this knowledge, bicistronic expression cassette, which includes a leader peptide and a second RBS between 5′-UTR and target gene, was explored and shown to be effective for increasing the expression activity of some native promoters (Mutalik et al. 2013; Zhao et al. 2016b). Then P_*dnaK*_ prolonged to the downstream sequence of initiation coden and carried additional optimal RBS and AS of R4a (P_*dnaK*_-2SD) was tested for its strength.

**Fig. 6:**
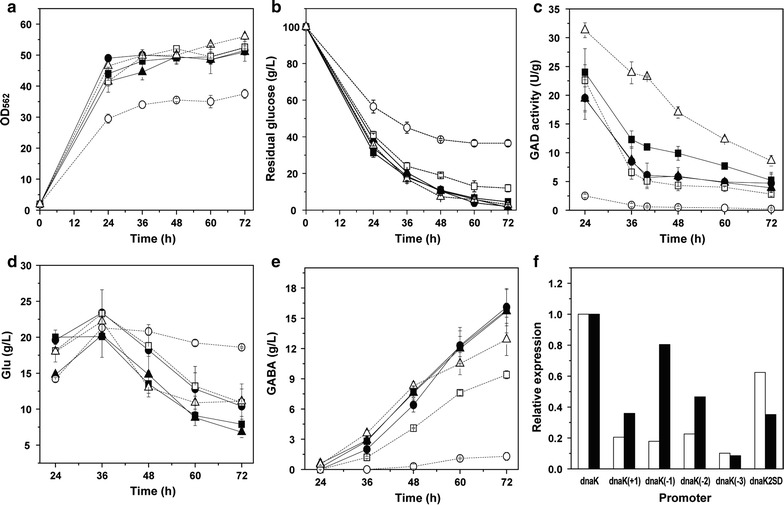
GABA fermentation of *gadB2*-expressing *C. glutamicum* strains under P_*dnaK*_ promoter with different length and bicistronic architecture. **a** Cell growth, **b** glucose consumption, **c** GAD activity, **d** Glu production, **e** GABA production, **f** *gadB2* transcription level. Squares P*dnaK*-B2, circles P*dnaK*(+1)-B2, triangles P*dnaK*(−1)-B2, empty squares in dotted line P*dnaK*(−2)-B2, empty circles in dotted line P*dnaK*(−3)-B2, empty triangles in dotted line P*dnaK*2SD-B2. White bars at 20 h, black bars at 40 h. Averages of three independent experiments are provided

The three *gadB2*-expressing strains under the prolonged P_*dnaK*_ promoters, i.e. P_*dnaK*(+1)_ prolonged to downstream 60 bp, P_*dnaK*(−1)_ prolonged to upstream 60 bp and P_*dnaK*(−2)_ prolonged to upstream 180 bp, as well as the strain under the bicistronic P_*dnaK*_ promoter (P*dnaK*2SD-B2) grew and consumed glucose in a similar manner with the strain P*dnaK*-B2 (Fig. 6a, b); meanwhile, the pH variation of these four strains was also similar to P*dnaK*-B2. However, the *gadB2*-expressing strain under the P_*dnaK*(−3)_ promoter that prolongs to upstream 240 bp grew and consumed glucose much slowly than P*dnaK*-B2, perhaps due to the reason that tedious fragment might increase the growth burden of bacteria. Meanwhile, its pH value during GABA conversion stage was significantly lower than other strains. The GABA production of P*dnaK*(+1)-B2 and P*dnaK*(−1)-B2 (about 16 g/L) was comparable to that of P*dnaK*-B2 (Fig. 6e) and similar amount of Glu (7–10 g/L) was remained (Fig. 6d), although their GAD activity and *gadB2* transcription level were somewhat lower than those of P*dnaK*-B2 (Fig. 6c, f), indicating the similar activity of P_*dnaK*(+1)_, P_*dnaK*(−1)_ and P_*dnaK*_ promoters for producing GABA. But the GABA production of P*dnaK*(−2)-B2 decreased by 40% and that of P*dnaK*(−3)-B2 decreased greatly to only 1.3 g/L; meanwhile, more Glu was remained and the total amount of Glu and GABA also decreased, likely due to the further decrease of GAD activity in these two strains, especially in P*dnaK*(−3)-B2, indicating the repression of P_*dnaK*_ as it extended to upstream 180 and 240 bp. Therefore, the activity of P_*dnaK*_ did not increase as P_*dnaK*_ region extended. Furthermore, although the GAD activity of P*dnaK*2SD-B2 was always higher than that of P*dnaK*-B2 (Fig. 6c), its *gadB2* transcription level (Fig. 6f) and GABA production (Fig. 6e) was lower than P*dnaK*-B2. Therefore, bicistronic architecture of P_*dnaK*_ was not beneficial for *gadB2* expression and GABA production in *C. glutamicum*.

### Coexpression of *gadB1*^mut^ and *gadB2* by optimal promoter and RBS for production of GABA

Even controlled by the robust promoter (P_*tacM*_) and RBS sequence (R4a), expression of *gadB2* only was not sufficient for converting all the synthesized Glu into GABA and approximately 5 g/L Glu was remained at the end of fermentation. Therefore, another GAD gene, *L. brevis gadB1*, was co-expressed with *gadB2*, both under the robust promoter of P_*tacM*_ and RBS sequence of R4a. Considering that as the active pH range of GadB1 was broadened to near-neutral pHs after mutagenesis, GABA production increased, especially in *gadB1*^T17I/D294G/E312S/Q346H^-expressing strain (Shi et al. 2014), this *gadB1* mutant (*gadB1*^mut^) was expressed here instead of wild-type *gadB1*.

**Fig. 7:**
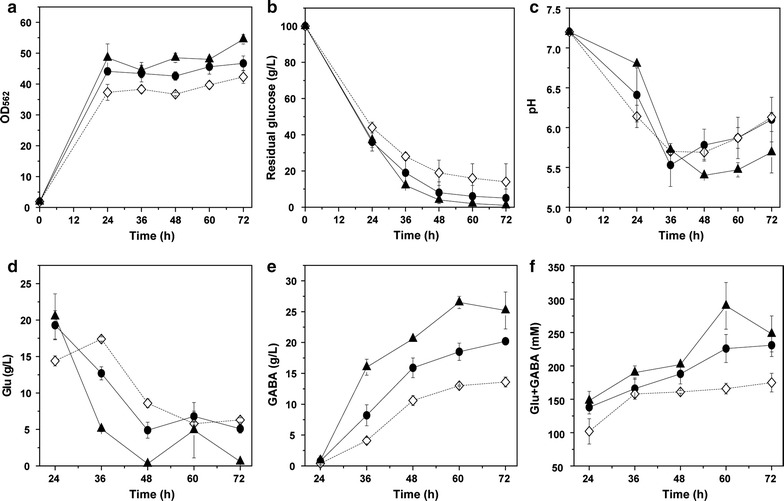
GABA fermentation of *gadB2*, *gadB1*^mut^ and *gadB2*-*gadB1*^mut^ expressing *C. glutamicum* strains under P_*tacM*_ promoter and R4a RBS sequence. **a** Cell growth, **b** glucose consumption, **c** pH variation, **d** Glu production, **e** GABA production, **f** total amount of Glu and GABA. Circles R4a-B2, empty diamonds in dotted line R4a-B1^mut^, triangles R4a-B2B1^mut^. Averages of three independent experiments are provided

The *gadB1*^mut^-expressing strain R4a-B1^mut^ grew and consumed glucose a little slowly, while the *gadB2*–*gadB1*^mut^-coexpressing strain R4a-B2B1^mut^ grew and consumed glucose a little faster than the *gadB2*-expressing strain R4a-B2 (Fig. 7a, b). The pH value of R4a-B1^mut^ varied similarly with that of R4a-B2, whereas the pH of R4a-B2B1^mut^ varied a little differently and increased to a lower level at the later stage of fermentation (Fig. 7c). The GAD activity of R4a-B1^mut^ (49.7 ± 1.8 U/g DCW) and R4a-B2B1^mut^ (127 ± 18 U/g DCW) was 55% and 3.0-fold higher than that of R4a-B2 (32.0 ± 2.5 U/g DCW), respectively. Compared with R4a-B2, more GABA (25.2 ± 3.0 g/L) was produced and nearly no Glu (0.6 ± 0.2 g/L) was remained in R4a-B2B1^mut^, whereas in R4a-B1^mut^, less amount of GABA and similar amount of Glu were produced (Fig. 7d, e). Meanwhile, the total amount of Glu and GABA of R4a-B2B1^mut^ was a little higher, whereas that of R4a-B1^mut^ was a little lower than that of R4a-B2 (Fig. 7f). Furthermore, in R4a-B2B1^mut^, the highest amount of GABA (26.5 ± 1.0 g/L) and the highest amount of both Glu and GABA (290 ± 35 mM) were obtained at 60 h and decreased thereafter, indicating the decomposition and consumption of GABA and Glu after 60 h, likely due to the exhaust of glucose. Therefore, coexpression of *gadB1*^mut^ and *gadB2* under the optimal P_*tacM*_ promoter and R4a RBS sequence was effective for production of GABA in *C. glutamicum*.

## Discussion

This study aims to ascertain an optimal combination of promoter and RBS sequence applying for the expression of GAD gene(s) and production of GABA in *C. glutamicum*. The expression level of heterologous genes has been illustrated to be influenced by multiple factors, including gene dosage, promoter strength, secondary structure of mRNA and RBS sequence (Stenstrom et al. 2001; Salis et al. 2009). To obtain the optimal expression element, successive investigation of RBS sequences and promoters was conducted.

For all the RBS sequences tested in this study, their strength for translating GadB2 and producing GABA was dramatically different (Fig. 1c, b). These RBS sequence can be used to translate protein in *C. glutamicum* at different levels. Two RBS sequences stronger than the frequently used RBS sequence of R, i.e. R4 and R6, were obtained here. Meanwhile, R4 showed the highest strength, whereas R9 and R3 showed the lowest strength. Similarly, the RBS sequence of R4 (GAAAGGAGA) was reported to exhibit higher expression level of NhhBA and specific activity of NHase in *C. glutamicum* (Kang et al. 2014). However, the RBS sequence of R3 (GAAAGGCGA) generated a slightly lower NhhBA level and NHase activity than R4, whereas here it resulted in the greatly decreased GAD activity and GABA production. In addition, the RBS sequence of R9 with AS of 8 nt (GAAAGGAGGtttggaca) was reported to be a strong RBS for expressing *vio* genes and producing violacein in *C. glutamicum* (Sun et al. 2016), meanwhile, the anti-SD sequence at the 3′-end of the 16S rRNA of *C. glutamicum* was described as 5′-CCUCCUUUC-3′ (Martín et al. 2003), whereas in our study R9 was too weak to express GadB2 and produce GABA. Therefore, the translation efficiency of different proteins through the identical RBS sequence of R3 or R9 may be dramatically different. However, the strong RBS sequences of R (AGAAGGAG) and R7 (GAAAGGA) used previously (Shi et al. 2013; Zhang et al. 2015b) were also effective for translating GadB2 and producing GABA. Besides RBS sequence, the AS between RBS and translational start codon also influenced GadB2 activity and GABA production significantly, with the AS of 6 nt the most efficient one (Fig. 2e). This is in consistent with previous study on the spacer length of *C. glutamicum* by transcriptome analysis, which showed 6 nt the maximum number (Pfeifer-Sancar et al. 2013). The RBS sequence and AS determine the affinity and accessibility of ribosome to RBS. At the beginning of translation, ribosome directly binds to RBS. A weaker secondary structure, i.e. fewer base pairs at RBS, can strengthen the accessibility and affinity of ribosome to RBS and thus improve the translation efficiency (Isaacs et al. 2004). Here, through optimizing RBS sequence and AS for translating GadB2, GAD activity increased by 156% and GABA production increased by 82% compared to the classic strong RBS of R and AS of 7 nt.

The strength of promoters tested in this study was also dramatically different as expressing *gadB2* and producing GABA (Figs. 3, 4, 5). These promoters with different strength can be applied to provide different level of gene expression in *C. glutamicum*. P_*tacM*_ showed the highest strength here, followed by P_*dtsR*_ and P_*dnaK*_, whereas P_*ilvE*_, P_*dnaJ*_, P_*sufR*_, P_*cg1417*_ and P_*gapA*_ showed the lowest strength. It is regrettable that none of the native promoters is stronger than P_*tacM*_. Considering that GABA is only synthesized after 24 h, *gadB2* expression shall be enhanced at stationary phase. The transcription level of *gadB2* under σ^B^-recognized promoters and stress response promoters, i.e. P_*hmp*_, P_*pqo*_, P_*ilvE*_, P_*cg1417*_, P_*dnaK*_, P_*clpB*_, P_*trxB1*_, P_*clgR*_ and P_*sufR*_, actually increased at GABA fermentation stage (Figs. 4d, 5d), but GAD activity and GABA production did not increased accordingly, perhaps due to incorrect fold or inactivation of GadB2 under certain stress. However, the reasons remain to be researched. The sigma factors for recognizing these promoters have been reported previously. P_*hmp*_, P_*pqo*_, P_*gapA*_, P_*ilvE*_ and P_*cg1417*_ are σ^B^-dependent (Larisch et al. 2007; Pátek and Nešvera 2011). P_*dnaK*_ is recognized by σ^A^, σ^E^ and σ^H^, while P_*clgR*_ be recognized by σ^E^ and σ^H^ (Šilar et al. 2016). P_*clpB*_ is depended on σ^M^ and σ^H^ (Ehira et al. 2009), while P_*trxB1*_ and P_*sufR*_ are σ^H^-specific (Dostálová et al. 2017). Even with prolonged length and bicistronic architecture, the strength of P_*dnaK*_ did not enhance (Figs. 5e, 6), although bicistronic expression archecture of 12 genes of *C. glutamicum* had been proven to be more efficient than monocistronic expression part (Zhao et al. 2016b). Despite this, several novel promoters that were as strong as P_*tuf*_ and P_*cspB*_, i.e. P_*dnaK*_, P_*dtsR*_, P_*odhI*_ and P_*clgR*_, were found in this study. P_*tuf*_ and P_*cspB*_ have been generally used as strong promoters for enhancing gene expression in *C. glutamicum* (Ravasi et al. 2012; Pátek et al. 2013; Vogt et al. 2015; Man et al. 2016). However, the widely applied strong promoter P_*sod*_ (Becker et al. 2011; Lee 2014; Kim et al. 2015; Man et al. 2016) showed very weak ability for expressing *gadB2* and producing GABA. P_*cg3141*_ (P_*hmp*_) which exhibited the highest inducibility for expression of a reporter, sfGFP during the transition phase between exponential and stationary phases in *C. glutamicum* (Kim et al. 2016) also showed much weak ability for GAD activity and GABA production. Therefore, all the native promoters tested here did not work as well as the strong synthetic promoter P_*tacM*_, partially due to the complex regulation of these native promoters by transcriptional regulators, such as regulation of P_*gapA*_ by GlxR and SugR, regulation of P_*dtsR1*_ by GlxR, repression of P_*dnaK*_, P_*clpB*_ and P_*clgR*_ by HspR, repression of P_*gdh*_ by GlxR, AmtR, FarR and ArgR, repression of P_*sufR*_ by SufR (Schroder and Tauch 2010). Recently, the strong synthetic promoter P_H36_ was employed for producing single-chain variable fragment of antibody (Yim et al. 2014), GABA (Choi et al. 2015) and 5-aminovaleric acid (Shin et al. 2016), while P_H30_ was proven to be more suitable than P_H36_ for the production of cadaverine in *C. glutamicum* (Oh et al. 2015). Therefore, synthetic promoters will be tested for *gadB2* expression and GABA production in the future.

Finally, two GAD genes, i.e. *gadB2* and *gadB1*^mut^, were co-expressed in *C. glutamicum*, both under the optimal P_*tacM*_ promoter and R4a RBS sequence. GAD activity increased greatly by 3.0-fold; consequently, GABA production increased to more than 25 g/L and all Glu was converted to GABA (Fig. 7d, e). However, the GABA production was not high enough. The GABA titer here was somewhat lower than that of GADΔ*pknG* (Okai et al. 2014) and recombinant *C. glutamicum* strain harbouring pHGmut (Choi et al. 2015) which expressed *E. coli* GAD and much lower than that of GABA6C and GABA6F (Jorge et al. 2017) whose putrescine pathway was engineered (Table 3). The GABA volumetric productivity here was comparable to that of strains reported previously with GAD activity and significant lower than that of strains with engineered putrescine pathway. In addition, the GABA yield on glucose here was lower than that of GADΔ*pknG* but comparable to that of other recombinant *C. glutamicum* strains. Considering that more GABA will be produced if the Glu production is high enough, fed-batch fermentation of R4a-B2B1^mut^ will be carried out in the future.

**Table 3:** GABA production in several recombinant *C. glutamicum* strains

Strains	GAD	GAD ΔpknG	Harbouring pHGmut	R4a-B2	R4a-B2B1^mut^	GABA6C	GABA6F
Cultivation in	Shake flask		Fed-batch	Shake flask		Fed-batch	
Pathway	GAD		GAD	GAD		Putrescine	
Cultivation time (h)	120	120	72	72	60	64	69
GABA titer (g/L)	13.1 ± 0.5	31.2 ± 0.4	38.6 ± 0.9	20.2 ± 0.3	26.5 ± 1.0	59.7	63.2
GABA volumeric productivity (g/L/h)	0.108	0.259	0.536	0.281	0.442	1.34	1.13
GABA yield on glucose (g/g)	0.156	0.511	0.320	0.212	0.269	0.24	0.24
References	Okai et al. (2014)		Choi et al. (2015)	This work		Jorge et al. (2017)	

## Abbreviations

GAD: glutamate decarboxylase
Glu: l-glutamate
GABA: γ-aminobutyric acid
RBS: ribosomal binding site
AS: aligned spacing
LAB: lactic acid bacteria
Lys: l-lysine
SD: Shine–Dalgarno
*gadB1*^mut^: *gadB1* ^T17I/D294G/E312S/Q346H^
LB: Luria–Bertani
CGMCC: China General Microbiological Culture Collection
LBG: LB supplemented with 5 g/L glucose
P_*dnaK*(+1)_: P_*dnaK*_ with the downstream 60 bp
P_*dnaK*(−1)_: P_*dnaK*_ with the upstream 60 bp
P_*dnaK*(−2)_: P_*dnaK*_ with the upstream 180 bp
P_*dnaK*(−3)_: P_*dnaK*_ with the upstream 240 bp
DCW: dry cell weight
RT-PCR: real-time PCR
Ct: cycle threshold
(A/G)_3_: AAA, AAG, AGA, AGG, GAG, GGG, GGA, GAA
H: high
M: medium
L: low
P_*dnaK*_-2SD: P_*dnaK*_ prolonged to the downstream sequence of initiation coden and carried additional optimal RBS and AS of R4a
R4: GAAAGGAGA
R9: GAAAGGAGG
R3: GAAAGGCGA
R: AGAAGGAG
R7: GAAAGGA
